# Overexpression of the alfalfa *WRKY11* gene enhances salt tolerance in soybean

**DOI:** 10.1371/journal.pone.0192382

**Published:** 2018-02-21

**Authors:** Youjing Wang, Lin Jiang, Jiaqi Chen, Lei Tao, Yimin An, Hongsheng Cai, Changhong Guo

**Affiliations:** Key Laboratory of Molecular Cytogenetics and Genetic Breeding of Heilongjiang Province, College of Life Science and Technology, Harbin Normal University, Harbin City, Heilongjiang Province, People’s Republic of China; National Taiwan University, TAIWAN

## Abstract

The WRKY transcription factors play an important role in the regulation of transcriptional reprogramming associated with plant abiotic stress responses. In this study, the WRKY transcription factor *MsWRKY11*, containing the plant-specific WRKY zinc finger DNA–binding motif, was isolated from alfalfa. The *MsWRKY11* gene was detected in all plant tissues (root, stem, leaf, flower, and fruit), with high expression in root and leaf tissues. *MsWRKY11* was upregulated in response to a variety of abiotic stresses, including salinity, alkalinity, cold, abscisic acid, and drought. Overexpression of *MsWRKY11* in soybean enhanced the salt tolerance at the seedling stage. Transgenic soybean had a better salt-tolerant phenotype, and the hypocotyls were significantly longer than those of wild-type seeds after salt treatment. Furthermore, *MsWRKY11* overexpression increased the contents of chlorophyll, proline, soluble sugar, superoxide dismutase, and catalase, but reduced the relative electrical conductivity and the contents of malonaldehyde, H_2_O_2_, and O_2_^-^. Plant height, pods per plant, seeds per plant, and 100-seed weight of transgenic *MsWRKY11* soybean were higher than those of wild-type soybean, especially OX2. Results of the salt experiment showed that *MsWRKY11* is involved in salt stress responses, and its overexpression improves salt tolerance in soybean.

## Introduction

Soybean is an important economic crop that has a high protein content and is better than any of the other common vegetable sources of protein; and it provides a vital source for human food, cooking oil, animal feed [1,2]. In addition, soybean oil is also used as a fuel source [3] and even as a source for medicines for its anticancer properties [4]. As a legume, soybean can effective improve nitrogen content in soil by fixing atmospheric nitrogen [5]. Soybean is considered a moderately salt-tolerant crop, but its productivity is critically affected by soil salinity because the germination rate and seed-setting rate decrease under salt stress [6]. In recent years, soil salinization has been rapidly becoming a serious problem, as 20% of cultivated land and 33% of irrigated land are salt-affected and degraded. The land available for agricultural crops has been decreasing every year [7]. The identification and characterization of salt tolerance genes is a crucial step for obtaining salt-tolerant soybean varieties, and transgenic technology is an important means for engineering salt-tolerant soybean lines.

Stress-inducible transcription factors (TFs) are important regulators of the stress response. They bind the *cis-*acting element to activate gene expression in response to stress, eventually protecting against or reducing damage to plants. Previous studies have reported increased plant salt tolerance due to the overexpression of stress-inducible TFs, such as CBF1/DREB1B, CBF2/DREB1C, DREB1A, DREB2A [8–12], NACs [13,14], bZIPs [15], MYBs [16], and WRKYs [17]. These TFs play a key role in activating the expression of various downstream genes, as well as in effectively protecting plants from salt stress.

WRKY transcription factors are major contributors that play an important role in survival of plants during environmental stress [18]. The *WRKY* family includes one or two domains, which is composed of about 60 amino acids. It has the conserved amino acid sequence WRKYGQK at the N-terminus and a C_2_H_2_ (Cx4-5Cx22-23HxH) or C_2_HC (Cx7Cx23HxC) zinc finger motif at the C-terminus [19,20]. WRKY TFs can regulate the plant hormone signal transduction pathway in the stress response, and also can activate or inhibit the expression of downstream genes through binding to the W-box motif (TTGAC/T) [21]. WRKY-type TFs are involved in multiple aspects of plant growth, development, and stress response. In recent years, an increasing number of WRKY TFs has been found to be involved in plant responses to abiotic stresses [22,23]. For example, the soybean gene *GmWRKY21* confers cold tolerance, whereas *GmWRKY54* increases tolerance to salt and drought stress [24]. Two wheat *WRKY* genes, *TaWRKY2* and *TaWRKY19*, contribute to salt and drought tolerance [25], whereas *Arabidopsis WRKY8* reacts antagonistically with *VQ9* to mediate salt stress responses [26]. Maize *ZmWRKY58* enhances drought and salt tolerance [27], and *Brassica campestris BcWRKY46* enhances tolerance to freezing, abscisic acid (ABA), salt, and dehydration stresses [28]. Cotton *GhWRKY68* increases the resistance to drought and salt and affects several physiological indices [29]. Overall, these studies elucidated that *WRKY* genes are broadly involved in plant resistance to abiotic stresses.

Physiological traits are important indicative indexes of abiotic resistance in plants. Additionally, salt stress can induce oxidative stress by continuously producing reactive oxygen species (ROS) [30]. SOD and CAT can remove ROS from the body to protect the enzyme system [31,32]. Relative electrical conductivity reflects the state of the plant membrane system, and MDA content denotes the degree of damage to the plant plasma membrane [33]. Soluble sugar and proline maintain osmotic balance as penetrating agents [34]. Currently, studies use physiological indicators as a standard for plant stress resistance analysis. *ThWRKY4* overexpression increases the tolerance of *Arabidopsis* to salt, oxidative, and ABA stress; improves SOD and POD activity; reduces the levels of ROS; and decreases the rate of cell death [35]. Overexpression of *DgWRKY3* in transgenic tobacco plants has been found to result in higher activity of antioxidant enzymes, including those of SOD, POD, and CAT, under salt stress [36]. Under salt stress, transgenic *DgWRKY5-*expressing chrysanthemum had higher activities of SOD, POD, and CAT enzymes than the WT, whereas the accumulation of H_2_O_2_, O_2_^−^, and MDA was decreased [37].

Alfalfa (*Medicago sativa*), a major forage legume cultivated worldwide, is resistant to various abiotic stresses, including salinity [38]. In alfalfa, only a small number of WRKY TFs has been isolated and characterized. *Medicago Sativa* L. Zhaodong is a highly salt-resistant species; it shows normal growth under 400 mM NaCl treatment. The sequence information of the Zhaodong alfalfa transcriptome under salt stress showed that *MsWRKY11* is up-regulated. In addition, a protein sequence analysis showed that the similarity of GmWRKY65 and MsWRKY11 was 71%, and this difference may be one of the reasons that the salt tolerance of Zhaodong alfalfa is stronger than that of soybean. Therefore, we chose Zhaodong alfalfa as the research material and cloned the *MsWRKY11* gene.

In the present study, the role of this gene in the plant response to abiotic stresses such as salinity, alkalinity, cold, ABA, and mannitol was examined and confirmed. To test whether *MsWRKY11* contributes to the soybean response to salt stress, we transformed this gene into soybean and investigated salt stress tolerance in the transformed soybean seedlings, salt-resistant phenotypes, and physiological characteristics. The results of this study will help to elucidate the role of *MsWRKY11* in the response to salt stress and provide a novel gene target for genetic engineering of salt-tolerant soybean lines.

## Materials and methods

### Cloning and sequencing of the *MsWRKY11* gene

*MsWRKY11* was cloned according to the sequence information of the transcriptome of the alfalfa cultivar Zhaodong under salt and temperature stress. Total RNA was isolated from alfalfa seedlings treated with NaCl solution using an RNA extraction kit (TianGen Biotech, Beijing, China) according to the manufacturer’s protocol. The RNA was reverse-transcribed into cDNA using a PrimeScript RT reagent kit (TaKaRa, Shiga, Japan) according to the manufacturer’s instructions. Reaction volumes (20 μL) contained 1 μL of Oligo_20_, 10 μL of RNA, 1 μL of RNase-free double-distilled water, 4 μL of 5× RT buffer, 2 μL of dNTP mix, 1 μL of RNase inhibitor, and 1 μL of reverse transcriptase. The amplification was run at 30°C for 30 min, 42°C for 20 s, 99°C for 5 min, and 4°C for 5 min (GeneAmp PCR System 9700, USA).

The cloning primers were designed according to the sequence information of the alfalfa transcriptome under salt stress by using Primer 5.0 software (primer 1: 5′-CTACCGGATCTACAACCATTCTAGAGC-3′, primer 2: 5′-CGAGCTCTCAAAGAGGCTGAGATAT-3′). The cDNA was diluted tenfold and used as the PCR template, and the *MsWRKY11* gene was amplified in 50 μL reactions containing 5 μL of cDNA, 5 μL of 10× dNTP mix, 4 μL of primer mix (primers 1 and 2), 0.2 μL of Ex Taq DNA polymerase (5 U/μL), and 31.8 μL of RNase-free double-distilled water. The cycling protocol consisted of the initial denaturation at 95°C for 3 min; 35 cycles of denaturation at 94°C for 30 s, annealing at 59°C for 30 s, and elongation at 72°C for 1 min; final elongation at 72°C for 10 min; and extension at 16°C for 2 h (GeneAmp PCR System 9700). The DNA fragments were recovered from the agarose gel using a DNA Recovery Kit (TianGen Biotech).

**Fig 1:**
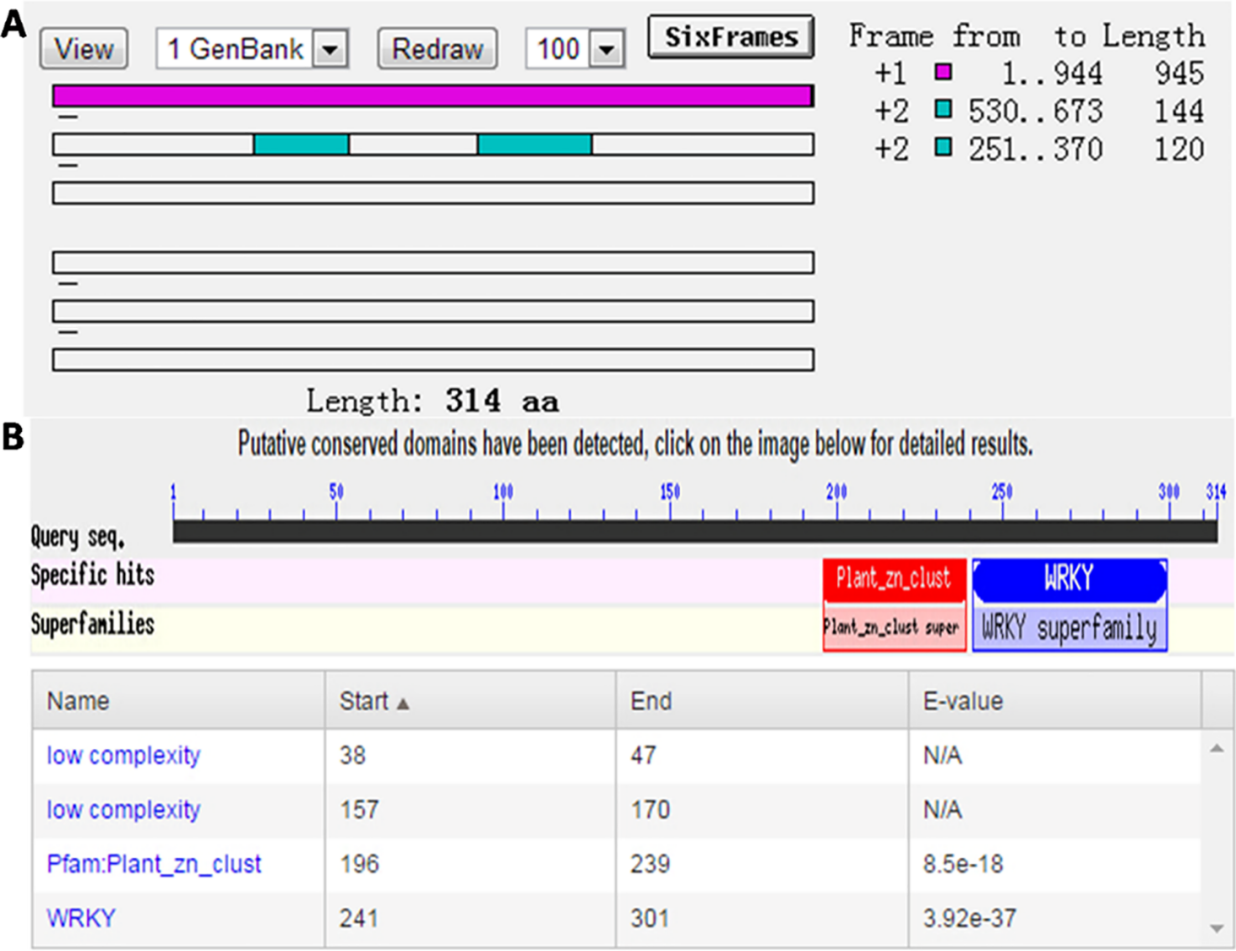
Open reading frame (ORF) prediction and *MsWRKY11* gene domains. (A) ORF Finder was used to predict the ORF of the *MsWRKY11* gene sequence. (B) The amino acid sequence was inferred by using DNAMAN software. The domain of the MsWRKY11 protein was obtained through NCBI protein BLAST search. MsWRKY11 has a zinc finger domain located at 196–239 aa and a WRKY domain located at 241–301 aa.

The PCR products were sent to Sangon Biotech Co., Ltd. (Shanghai, China) for sequencing. The amino acid sequence was inferred by DNAMAN software (Lynnon LLC, San Ramon, CA, USA). Sequences from other species were obtained from GenBank (https://www.ncbi.nlm.nih.gov/protein) and aligned with the sequence for *MsWRKY11* in ClustalX [39] (Fig 1). The neighbor-joining method was used to generate the phylogenetic tree. The protein sequences of MsWRKY11, GmWRKY65, GmWRKY11, and AtWRKY11 were analyzed using DNAMAN.

### Expression of *MsWRKY11* in alfalfa

#### Plant material and treatment conditions

Alfalfa seeds were germinated in pots with distilled water for 2 days in the dark; transferred to vermiculite with ½ Hoagland solution; and kept in a culture room for 4 weeks at 60% relative humidity, 22°C, and a photoperiod of 16 h light/8 h dark. Four-week-old seedlings were treated with ½ Hoagland solution containing salt (300 mM NaCl), alkali (0.1 M mixed alkaline solution of Na_2_CO_3_ and NaHCO_3_ in a ratio of 1: 2), or ABA (15 μM), or were subjected to cold (−4°C) or drought (simulated with 150 mM mannitol) stress for 0 (control), 1, 3, 6, 12, or 24 h. The harvested whole seedlings from each treatment were frozen in liquid nitrogen and stored at −80°C for RNA extraction and quantitative reverse transcription PCR (qRT-PCR) analysis.

#### qRT-PCR assays

First-strand cDNA from alfalfa was used for qRT-PCR assays for the expression pattern analysis of the response to abiotic stresses and tissue-specific expression analysis. Total RNA was extracted from the same group of samples that was used in the transcriptome analysis using an RNA extraction kit (TianGen Biotech). qRT-PCR primers were designed using Primer 5.0 software (5’-CTTGTTCTTCAAATCAACGT-3’ and 5’-AATTCGTGTTCCGGCGATAA-3’).

The reaction mixture (20 μL) contained 10 μL of SYBR Green RealTime PCR Master Mix (Toyobo, Osaka, Japan), 2 μL of cDNA template, and the forward and reverse primers at 0.5 μM each. The actin gene was used as the reference gene. In the salinity, alkalinity, cold, abscisic acid, and drought stress experiments, the Ct value of the actin gene was stable. The amplification was run in an ABI 7300 sequencer (USA) with the following cycling parameters: 94°C for 30 s, followed by 45 cycles at 94°C for 15 s, 55°C for 30 s, and 72°C for 30 s. qRT-PCR was performed in triplicate to confirm the accuracy of the results. All of the relative expression levels were log_2_-transformed.

### Construction of the plant expression vector pTF101.1-*MsWRKY11* and its transformation into soybean

Full-length cDNA of *MsWRKY11* was obtained from alfalfa, and pMD18-T:: *MsWRKY11* (constructed using the TaKaRa Reagent kit) and the expression plasmid pTF101.1-*MsWRKY11* were constructed (using the restriction endonucleases *Xba*I and *Sac*I). The *WRKY11* fragment and pTF101.1 plant expression vector were connected using ligase and transformed into *Escherichia coli* by the thermal stimulation method. Finally, the recombinant plasmid was transformed into *Agrobacterium* strain EHA101 using the freezing–thawing method. Soybean variety DongNong-50 was transformed using the EHA101 strain (containing the plant expression vector pTF101.1-*MsWRKY11*) by the *Agrobacterium*-mediated co-cultivation method. The protocol was previously described by Kim et al. [40–42].

### Growth conditions for transgenic soybean

Plastic pots (10 cm diameter × 15 cm high) containing 3 kg stroma (peat soil: vermiculite = 1:1, v/v) were used for planting. Three plants per pot were planted and kept in a culture room at 60% relative humidity and 25°C. Watering was performed every 4 days, and the light/dark condition was set to 16 h/8 h. Growth conditions were the same for all experiments.

### Detection of successfully transformed soybean plants by PCR

T_0_ soybean plants were identified by PCR. The cetyltrimethylammonium bromide (CTAB) method was used for isolation of small amounts of DNA from soybean leaves. Primers for the amplification of *MsWRKY11* were as follows: primer 3: 5’-TACCGGATCTACAACCATTCTAGAGC-3’ and primer 4: 5’-CGAGCTCTC AAAGAGGCTGAGATAT-3’. Each reaction mixture (10 μL) contained 1 μL of DNA template, 1 μL of 10× PCR buffer, 0.8 μL of dNTP mix (2.5 mmol/L), 0.8 μL of primer mix, 0.05 μL of rTaq DNA polymerase (5 U/μL), and 6.35 μL of RNase-free double-distilled water. The amplification of *MsWRKY11* was conducted under the following conditions: 95°C for 3 min; 34 cycles of denaturation at 94°C for 30 s, annealing at 59°C for 30 s, and elongation at 72°C for 30 s; final elongation at 72°C for 10 min; and final extension at 16°C for 2 h (GeneAmp PCR System 9700).

### qRT-PCR validation of transgenic lines

Total RNA was isolated from the leaves of transgenic and wild-type soybean plants using an RNA extraction kit (TianGen Biotech) following the manufacturer’s protocol. Data were normalized using the actin gene. The RNA was reverse-transcribed into cDNA using a PrimeScript RT reagent kit (TaKaRa). The qRT-PCR primers were the same as those used in the qRT-PCR analysis of alfalfa. Each 20 μL qRT-PCR reaction contained 10 μL of Thunderbird SYBR qPCR mix (Toyobo), 0.4 μL of 50× ROX reference dye, 1.6 μL of primer mix, 2 μL of diluted cDNA, and 6 μL of RNase-free double-distilled water. The reactions were subjected to an initial denaturation at 95°C for 10 min, followed by 40 cycles at 95°C for 15 s, 53°C for 30 s, and 72°C for 30 s. Amplification was followed by a melting curve analysis of amplified products according to the following protocol: 95°C for 15 s, 60°C for 20 s, and 95°C for 15 s (ABI 7300). Data from the qRT-PCR experiments were analyzed according to the 2^−∆∆Ct^ method [43]. Each sample was prepared in triplicate.

### Salt tolerance experiments

For the seed germination experiment, T_3_ homozygous transgenic soybean seeds and wild-type seeds were surface sterilized and placed on ½ Murashige and Skoog basal nutrient salts with B5 vitamins, supplemented with NaCl (0, 100, 200 mM) under a 16 h/8 h light/dark cycle at 25°C for 7 days. Images were taken at the end of each experiment, and hypocotyl length (the length from the cotyledon to the root tip) was recorded.

To examine the salt tolerance of soybean leaves, leaves of 4-week-old T_3_ homozygous transgenic lines (OX1, OX2, OX4) and wild-type plants were irrigated with a solution containing 200 mM NaCl for 7 days. Their leaves were collected and soaked in culture dishes that were filled with NaCl solution (200 mM) under a 16 h/8 h light/dark cycle and 25°C. All experiments were repeated thrice, and phenotypic variations in the leaves were recorded daily.

For the assessment of soybean plant tolerance to salt, 4-week-old seedlings of T_3_ homozygous transgenic lines (OX1, OX2, OX4) and wild-type plants (3 plants per pot) were irrigated with a solution containing 200 mM NaCl for 10 days. The control group was irrigated with the same volume of water. All of the plants were kept under a 16 h/8 h light/dark cycle at 25°C.

### Estimation of chlorophyll content

Fresh soybean leaves (0.1 g) were placed in 50 mL centrifuge tubes and extracted with 10 mL of 95% ethanol in the dark (24 h). The absorbance of the extract was measured using an ultraviolet spectrophotometer (UV-5100 Spectrophotometer, Shanghai, China) at 645 nm and 663 nm. Total Chl, Chl*a*, and Chl*b* content (mg/g fresh weight) were calculated as follows: [Chl*a*] = 12.72 × A_663_ − 2.59 × A_645_, [Chl*b*] = 22.88 × A_645_ − 4.67 × A_663_, and [Chl*a*+*b*] = 8.02 × A_663_ + 20.21 × A_645_ [44].

### Measurement of physiological parameters under salt stress

Four-week-old seedlings of T_3_ homozygous transgenic lines (OX1, OX2, OX4) and wild-type plants were exposed to 200 mM NaCl for 10 days and 0.1 g of leaf tissue was used to measure the activity of catalase (CAT) and superoxide dismutase (SOD); the content of free proline, soluble sugars, malondialdehyde (MDA), H_2_O_2_, and O_2_^-^; and the relative conductivity. CAT activity was assayed following Zhang et al. [45]; SOD activity, according to Beauchamp et al. [46]; MDA, according to Hodges et al. [47]; and proline and soluble sugars, according to Lrigoyen et al. [48]. Relative conductivity was assessed following Lutts et al. [49]; H_2_O_2_ content, as described by Mukherjee et al. [50]; and O_2_^-^ content, as described by Liu et al. [51]. All experiments were repeated thrice.

### Statistical analysis

Each physiological parameter measurement was repeated three times. Data were randomly selected and processed using SPSS 13.0 Data Editor. The differences between groups were processed using Duncan’s test and Excel was used to draw the chart. *p* < 0.05 was considered statistically significant.

### Agronomic character analysis

We randomly measured 30 strains each from three transgenic lines and wild soybean and the average was taken as the final result (Table 1). Agronomic traits included plant height, branches, pods per plant, seeds per plant, seed weight per plant, and 100-seed weight. The data were analyzed by one-way ANOVA using SAS9.0, and the *t*-test was used to evaluate the results.

**Table 1:** Agricultural yield characteristics of transgenic and wild-type soybean.

Lines	Plant height (cm)	Branches number	Pod number per plant	Seed number per plant	100-seed weight per plant (g)
WT	58.5 ± 3.5	5 ± 1.0	90 ± 15	180.5 ± 33	5.4
OX1	68.7 ± 3.2	4 ± 1.0	94 ± 16	197.7 ± 37	5.5
OX2	69.7* ± 5.2	5 ± 0.5	97.7* ± 16	201.2* ± 36	5.7*
OX4	68.0 ± 1.0	5 ± 2.0	95.0 ± 16	200.0 ± 38	5.5

Agricultural yield of T3 transgenic soybean and wild-type soybean. Agronomic traits included plant height, branches, pods per plant, seeds per plant, and 100-seed weight. The data were analyzed by one-way ANOVA using SAS9.0, and the t-test was used to evaluate the results.

* indicates significant difference with wild type at 0.05 level; WT is wild-type variety Dongnong 50. OX1, OX2, and OX4 are *MsWRKY11* over-expressing transgenic lines.

## Results

### Identification and sequence analysis of the *MsWRKY11* gene

Cloning primers were designed according to the sequence information of the alfalfa transcriptome under salt stress conditions, and the *MsWRKY11* gene was cloned by RT-PCR. The length of the gene is 945 bp, and it encodes 314 amino acids. Protein domain analysis showed that *MsWRKY11* has a zinc finger domain at 196–239 aa and the WRKY domain at 241–301 aa. These results clearly indicate that this gene belongs to the *WRKY* gene family (Fig 1).

### Homology of the WRKY11 sequence and phylogenetic tree of different plant species

**Fig 2:**
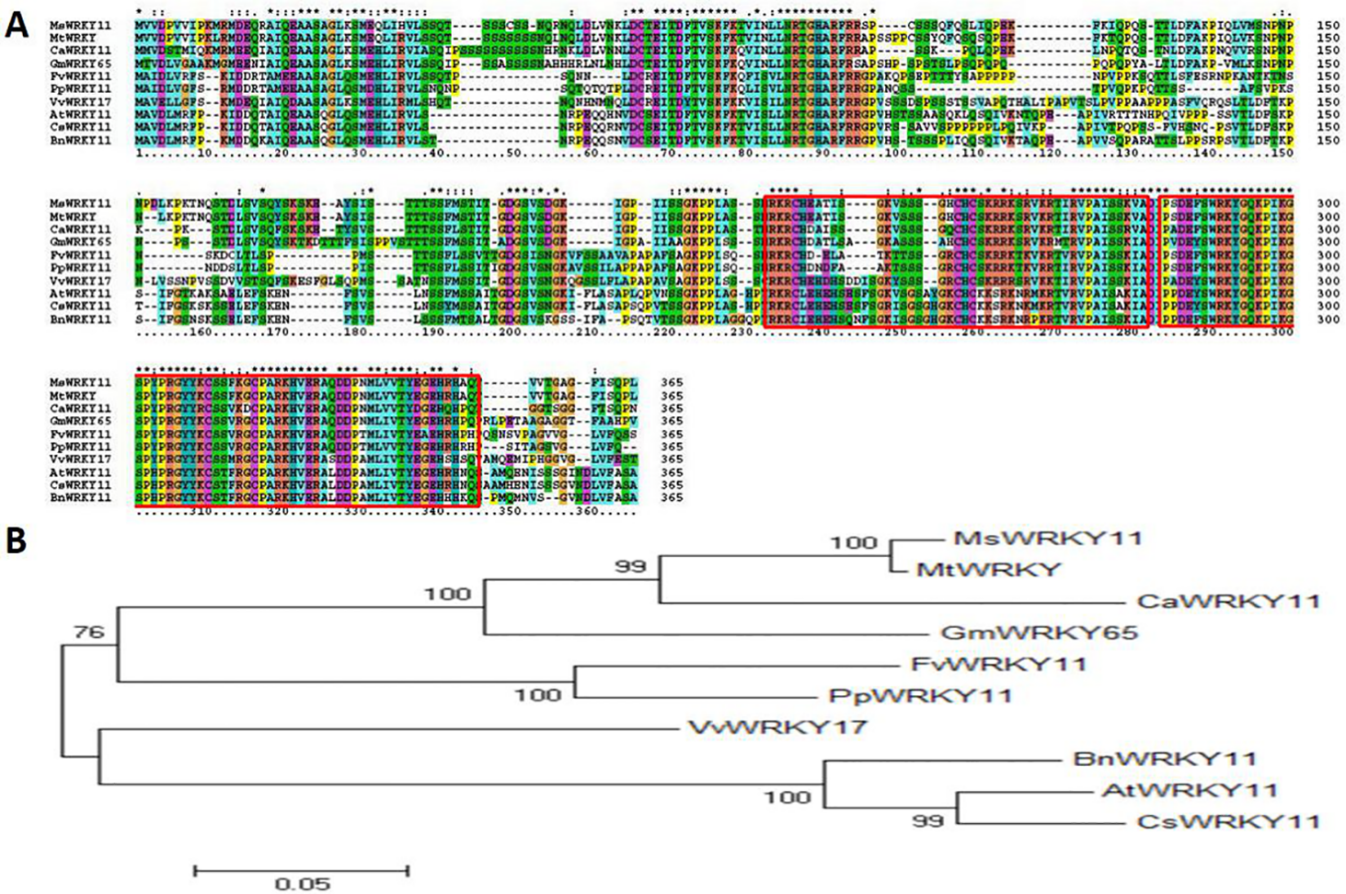
WRKY11 sequence homology analysis and phylogenetic tree based on the WRKY11 sequences of different species. (A) The amino acid sequence of the gene was deduced by DNAMAN software. (B) Phylogenetic analysis of *WRKY11* in different plants, including *Medicago sativa*, *Medicago truncatula*, *Cicer arietinum*, *Glycine max*, *Fragaria vesca*, *Prunus persica*, *Vitis vinifera*, *Brassica napus*, *Arabidopsis thaliana*, and *Cucumis sativus*. The neighbor-joining method was used to generate the phylogenetic tree.

Protein Clustalx analysis of MsWRKY11 with homologous proteins from various species, such as Leguminosae, Rosaceae, Vitaceae, Cruciferae, and Cucurbitaceae, showed that WRKY11s presented a highly conserved domain in experimental material species, including a zinc finger domain and a WRKY domain (Fig 1A). However, the core domain was also different in Rosaceae, Vitaceae, Cruciferae, and Cucurbitaceae species compared with Leguminosae (Fig 2). In Leguminosae plants, the protein sequence of MsWRKY11 revealed 95% identity with MtWRKY, 78% identity with CaWRKY11 and 71% identity with GmWRKY65. In the core domain, MsWRKY11 had 100% identity with MtWRKY (Fig 2A). In the zinc finger domain, GmWRKY11 had insertion of an amino acid compared with MsWRKY11, MtWRKY, and CaWRKY11. In the WRKY domain, CaWRKY11 and GmWRKY65 had a substitution of 5 amino acids compared with MsWRKY11. The protein sequence of GmWRKY11, MsWRKY11, AtWRKY11, and GmWRKY65 is shown in S1 Fig. The MsWRKY11 gene was homologous to AtWRKY11 in the Arabidopsis homologous gene, with an identity of 53%. The MsWRKY11 gene had the closest homology with GmWRKY65 in soybean, with an identity of 71% (S1 Fig).

### Expression traits of *MsWRKY11* in alfalfa

**Fig 3:**
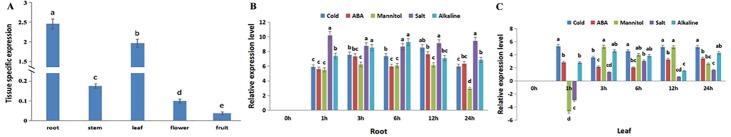
Quantitative reverse transcription-PCR (qRT-PCR) analysis of *MsWRKY11* expression patterns in *Medicago sativa*. (A) Tissue-specific expression of *MsWRKY11* in root, stem, leaf, flower, and fruit tissues. (B, C) The expression pattern of *MsWRKY11* in the root and leaf under abiotic stresses: salinity (300 mM NaCl); alkalinity (0.1 M alkaline solution of Na_2_CO_3_ and NaHCO_3_ in a 1:2 ratio); abscisic acid (ABA; 15 μM); drought (simulated with 150 mM mannitol); and cold (−4°C) for 1, 3, 6, 12, and 24 h. qRT-PCR was performed in triplicate to confirm the accuracy of the results. All of the relative expression levels were log_2_-transformed.

Four-week-old seedlings were used for qRT-PCR to elucidate the tissue-specific expression of *MsWRKY11* in alfalfa. The qRT-PCR analysis showed that *MsWRKY11* was highly expressed in the roots and leaves, and trace levels were detected in the stem, flower, and fruit (Fig 3A). To analyze the expression pattern of *MsWRKY11* under abiotic stresses, alfalfa plants were exposed to salinity, alkalinity, cold, ABA, and drought, and gene expression levels were measured in the roots and leaves using qRT-PCR (Fig 3B and 3C). In treatments with 300 mM NaCl, *MsWRKY11* was significantly upregulated in the leaves, but there was no significant change in the expression level in the root tissue. Similar patterns in the expression of *MsWRKY11* were observed with alkali, cold, drought, and ABA treatments in the root. In contrast, the expression of *MsWRKY11* in the leaves varied under different treatments. In treatments with 300 mM NaCl, the *MsWRKY11* transcript level was significantly reduced at 1 h, increasing subsequently and peaking at 6 h of the treatment. A similar expression pattern was found with drought treatments. Alkali treatment increased the transcript level, which reached the first peak at 3 h. This was then followed by a slight reduction in gene expression after 6 h and 12 h of treatment, but another upregulation followed, with the second peak at 24 h. The response of *MsWRKY11* in leaf tissue to cold stress and ABA followed a pattern similar to that under alkaline stress.

### Transformation of *MsWRKY11* into soybean

**Fig 4:**
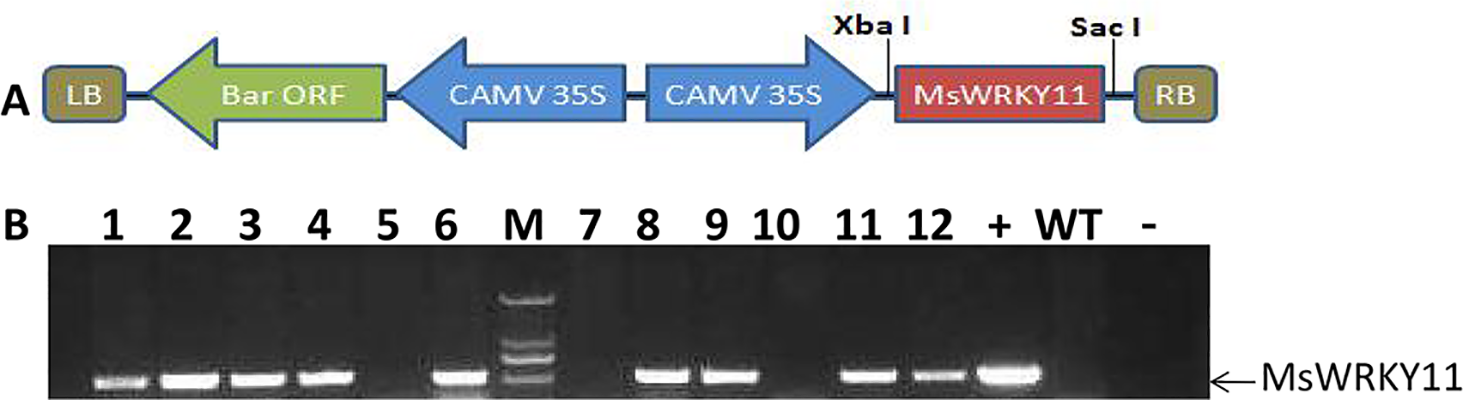
Construction of the plant expression vector and results of the PCR analysis of T_0_ transgenic *MsWRKY11* soybean. (A) Construction of the plant expression vector pTF101.1–*MsWRKY11*. pTF101.1 plasmid and pMD18T::*MsWRKY11* were digested using *Xba*I and *Sac*I, respectively. T4 ligation was employed to connect the products of the restriction, which were then transferred into *Escherichia coli* competent cells (DH5α). (B) PCR analysis of transgenic plants: positive transgenic lines and the positive control contain a 600 bp band; M: DL2000 DNA ladder; +: positive control;–: ddH_2_O; WT: wild-type soybean.

The expression vector pTF101.1, which contains the herbicide resistance gene *bar*, was used for transformation. *MsWRKY11* was digested at the *Xba*I and *Sac*I restriction enzyme sites. The cloning of *MsWRKY11* into the plant expression vector is shown in Fig 4A. We obtained 16 seedlings with glufosinate resistance, which were examined by PCR analysis using the *MsWRKY11* gene. Finally, we obtained 9 T_0_-positive transgenic lines (Fig 4B).

### qRT-PCR validation of transgenic lines

**Fig 5:**
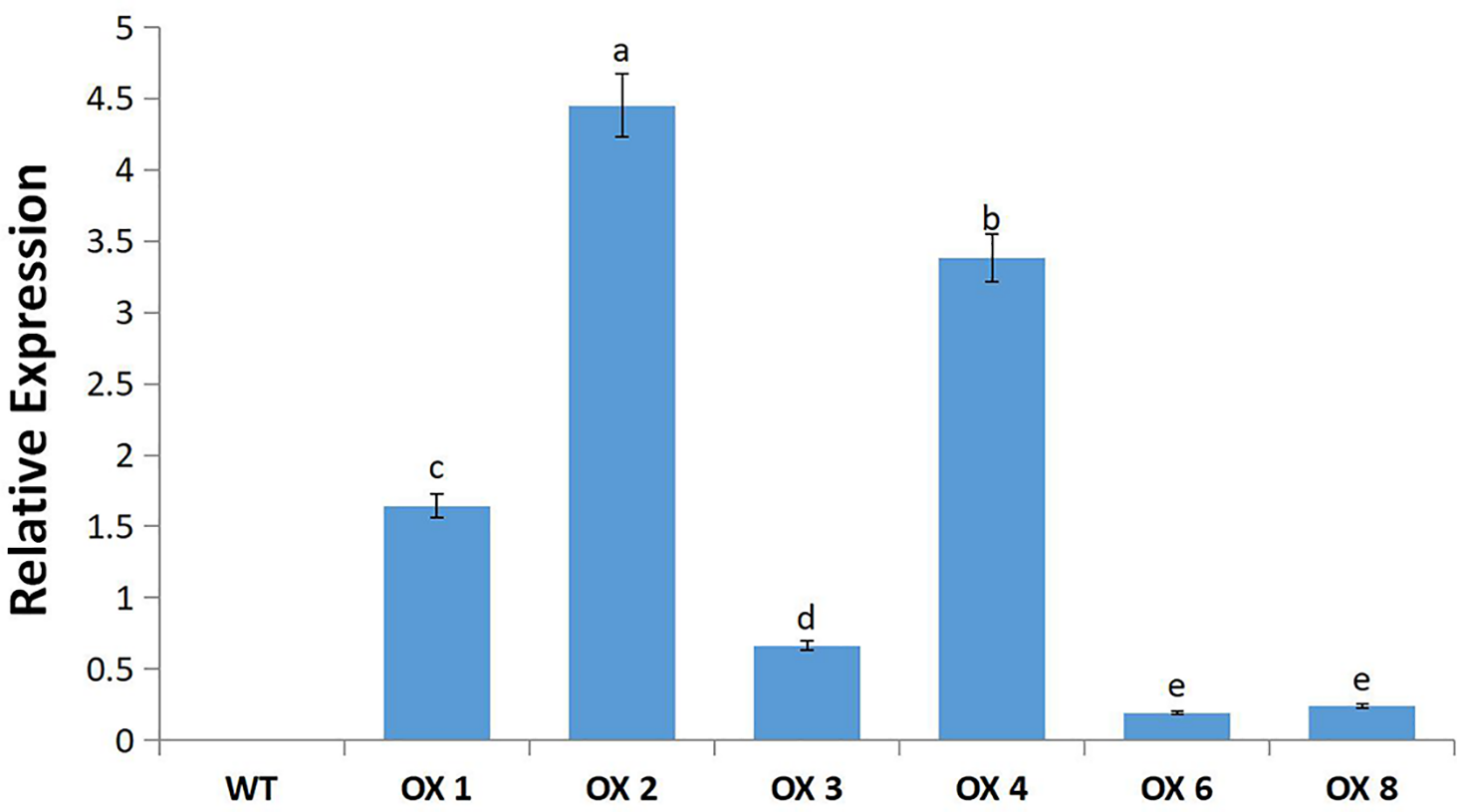
Expression level of *MsWRKY11* in T_0_ transgenic soybean plants. Four-week-old soybean leaves were used for qRT-PCR. Data from the qRT-PCR experiments were analyzed according to the 2^−∆∆Ct^ method. Vertical bars indicate standard deviation calculated from 3 replicates. Means denoted by the same letter do not differ significantly at *p* < 0.05.

*MsWRKY11* expression using qRT-PCR was confirmed in T_0_-positive transgenic soybean lines (OX1, OX2, OX4); the gene was not expressed in the wild type (Fig 5).

### Seed germination under salt stress

**Fig 6:**
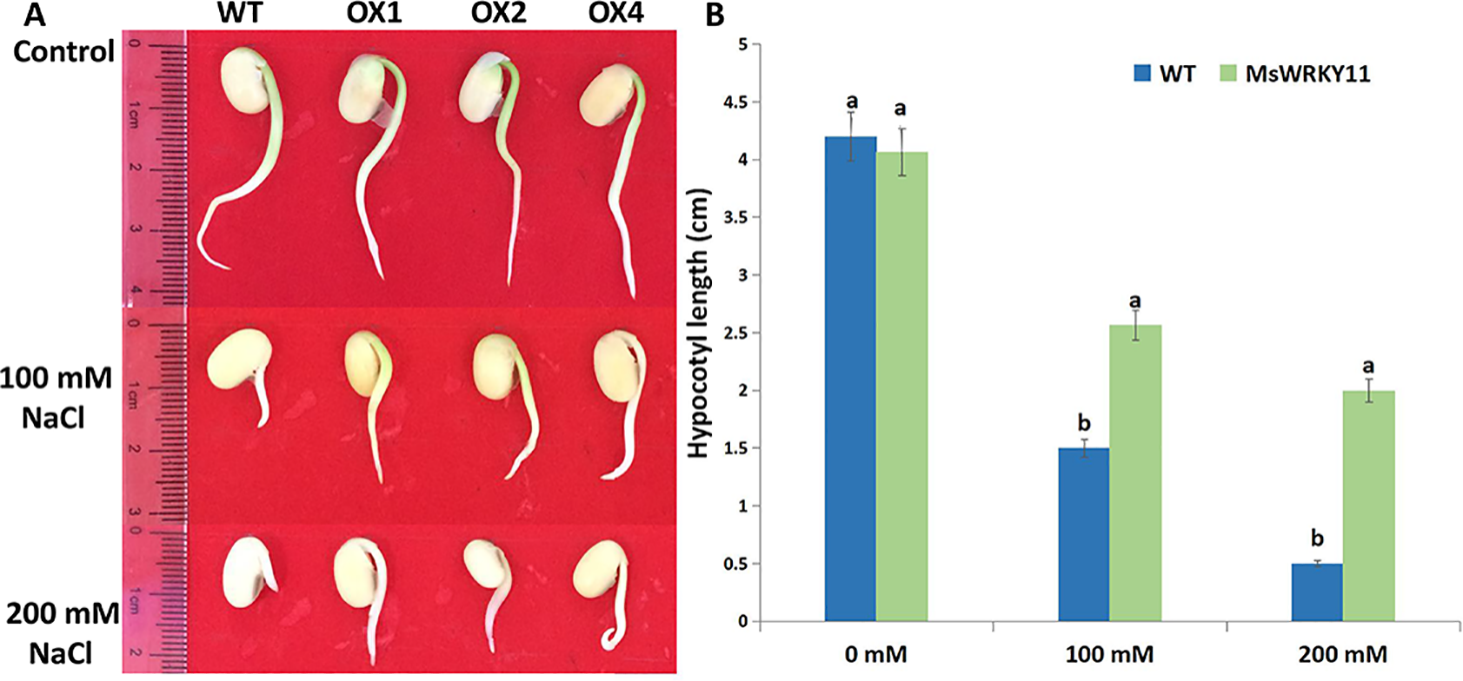
Seed germination under salt stress. (A) Germination of soybean seeds in RM solid medium containing 0 mM, 100 mM, and 200 mM NaCl. (B) Hypocotyl length of transgenic and WT seedlings.

To determine hypocotyl length in plants with overexpressed *MsWRKY11* under salt stress, seeds of T_3_, i.e., the homozygous transgenic progeny of the OX1, OX2, and OX4 lines, were germinated in medium containing different salt concentrations. In treatments with 100 mM NaCl, the hypocotyls were shorter in wild-type plants than in transgenic lines (Fig 6A). Under the salt concentration of 200 mM, hypocotyl length decreased but remained higher than that of the wild type (Fig 6B). These results indicate that overexpression of *MsWRKY11* could improve the germination rate and hypocotyl length in seedlings under salt stress.

### Phenotypic analysis of transgenic plants under salt stress

**Fig 7:**
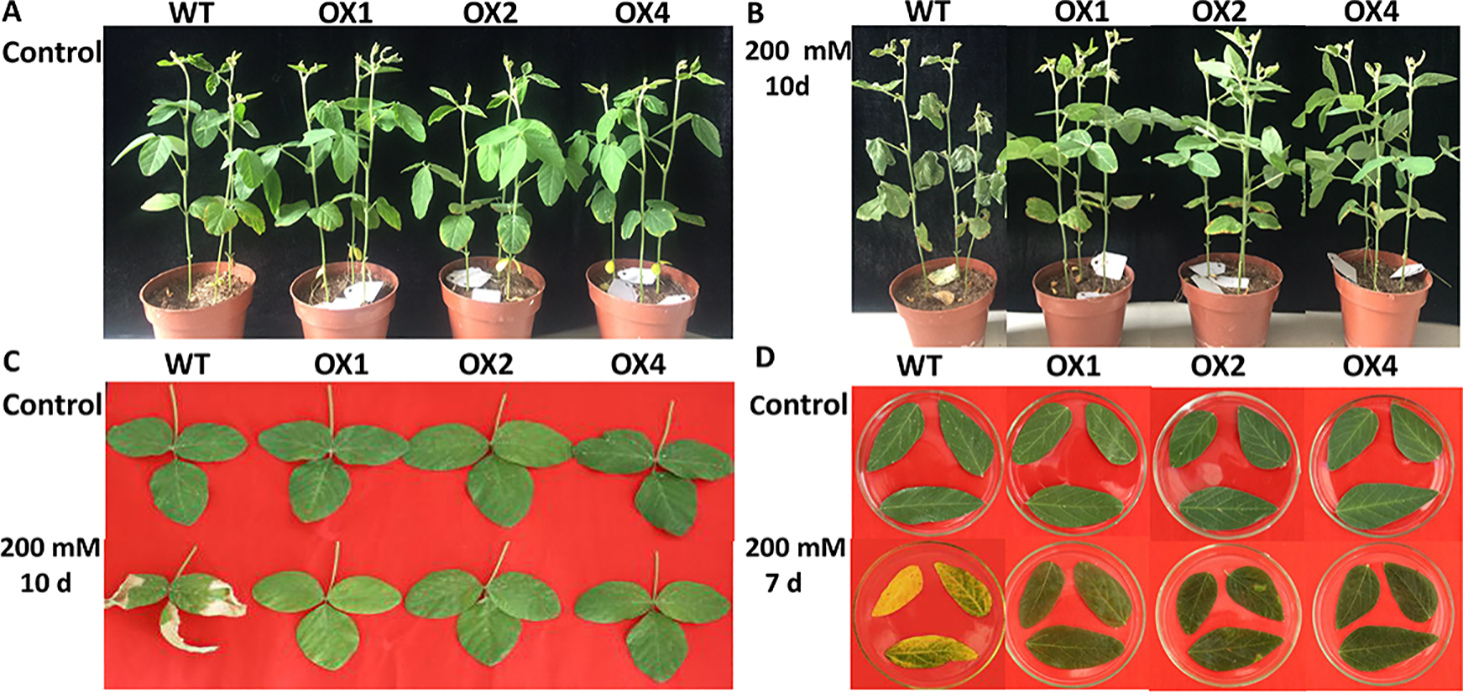
Phenotypic comparison chart of T_3_ transgenic and wild-type (WT) soybean plants under salt stress (200 mM NaCl). (A) Phenotype before salt stress. (B) Phenotype after 10 days of salt treatment. (C) Phenotype after 10 days of salt treatment. (D) Salt tolerance analysis of *MsWRKY11* transgenic soybean leaves. Leaves from the T_3_ transgenic lines OX1, OX2, and OX4 and the WT were processed for 7 days in culture dishes (200 mM NaCl).

The phenotypes of the 4-week-old T_3_ transgenic plants were determined under high salt stress (200 mM) (Fig 7A). After 10 days of treatment, the leaves of the wild type turned yellow and dropped. By contrast, few leaves of transgenic lines turned yellow, and plant growth remained vigorous (Fig 7B). It is evident that *MsWRKY11* overexpression in soybean improved the salt tolerance. Leaves removed from 4-week-old T_3_ transgenic plants were assayed under high salt stress (200 mM). The leaves of the wild-type soybean turned yellow after 7 days of salt treatment, whereas those of the transgenic soybean maintained their normal color (Fig 7D).

### Chlorophyll content of *MsWRKY11* transgenic soybean

**Fig 8:**
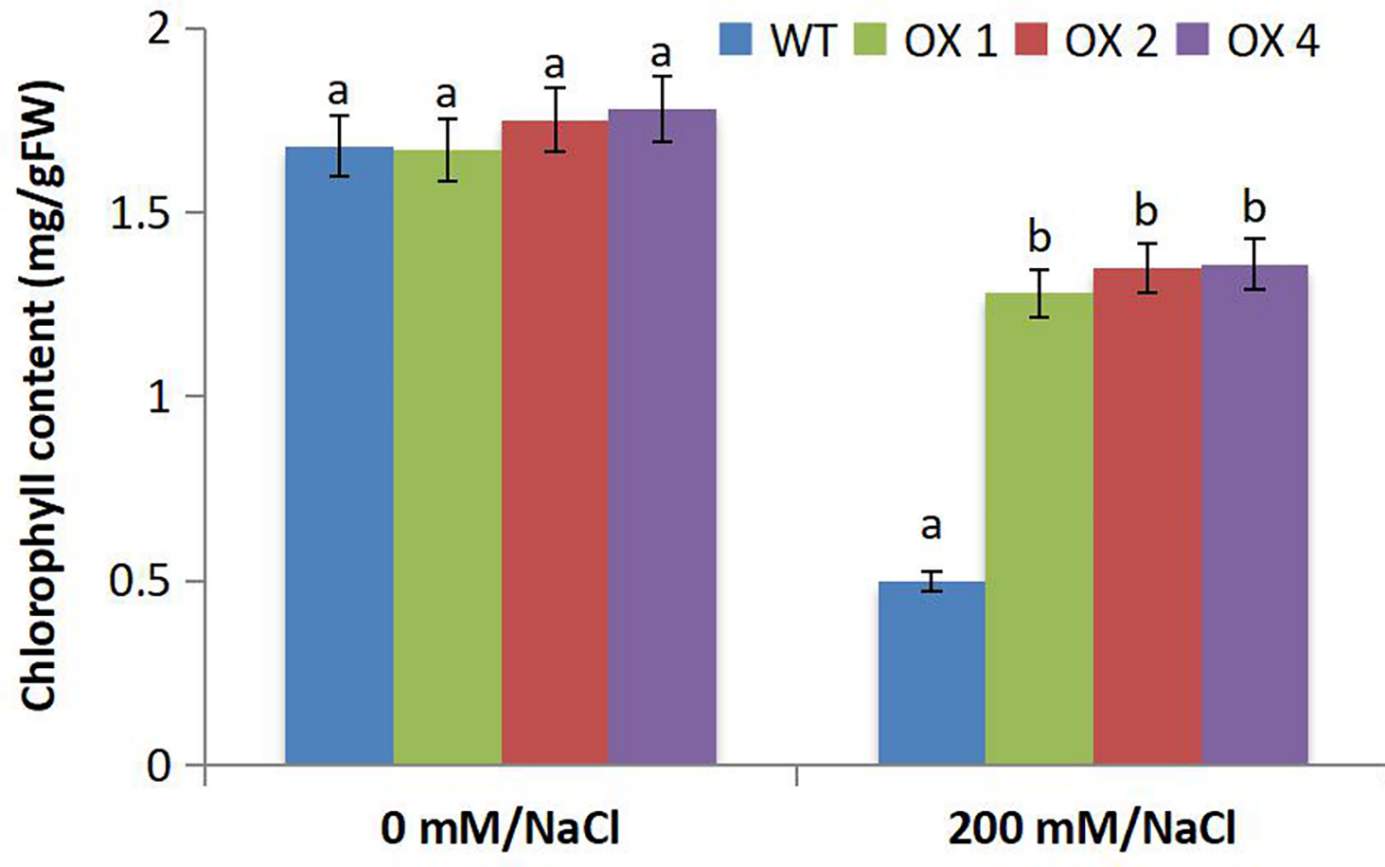
Chlorophyll content of T_3_ *MsWRKY11* transgenic lines OX1, OX2, and OX4. Chlorophyll (Chl) content was measured using 0.1 g of fresh soybean leaves and expressed as [Chl*a*], [Chl*b*], and [Chl*a*+*b*]. Vertical bars indicate the standard deviation calculated from 3 replicates. Means with the same letter do not differ significantly at *p* < 0.05.

Chlorophyll content in the leaves of T_3_ transgenic (OX1, OX2, and OX4) and wild-type plants was determined at the pod stage. The chlorophyll content in the leaves of transgenic lines was significantly higher than that in wild-type lines, suggesting that the degree of structural and functional damage of the chloroplast in transgenic soybean was lower than that of the wild type in saline environments (Fig 8).

### Physiological index analysis under salt stress

Salt treatment induced changes in plant physiological indexes, such as CAT and SOD activity; free proline, soluble sugar, MDA, H_2_O_2_, and O_2_^-^ content; and relative electrical conductivity. These indicators reflect the ability of plants to respond to salt stress. After 1 week of salt stress treatment (200 mM), we determined the physiological activity of T_3_ transgenic lines and the wild type.

**Fig 9:**
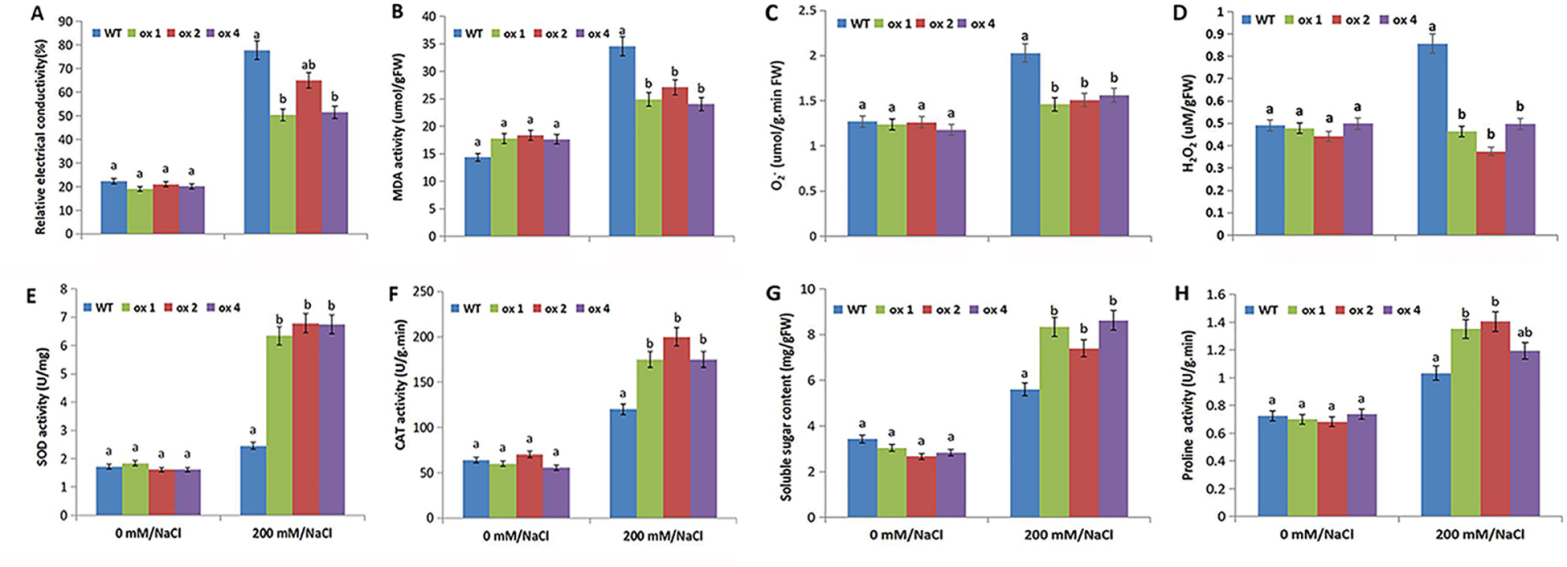
Analysis of physiological parameters between wild-type (WT) and transgenic T_3_ lines (OX1, OX2, OX4). (A) Relative electrical conductivity. (B) Malonaldehyde (MDA). (C) O_2_^-^. (D) H_2_O_2_. (E) Superoxide dismutase (SOD). (F) Catalase (CAT). (G) Soluble sugar. (H) Proline. All values were measured after 7 days of exposure to salt stress (200 mM NaCl). Vertical bars indicate standard deviation calculated from 3 replicates. Means denoted by the same letter do not differ significantly at *p* < 0.05.

The relative conductivity of the leaves in T_3_ transgenic lines was significantly lower than that of wild-type plants (Fig 9A). The MDA, H_2_O_2_, and O_2_^-^ contents increased in both transgenic and wild-type plants under salt stress, but the increase was greater in the wild-type plants than in transgenic lines (Fig 9B–9D). Similarly, the CAT, SOD, free proline, and soluble sugar levels increased in both transgenic and wild-type plants, but the increase was greater in transgenic plants than in the wild-type plants (Fig 9E–9H). Furthermore, the free proline and soluble sugar content of transgenic plants and wild-type plants increased under salt stress, but the increase was higher in transgenic plants (Fig 9G and 9H).

### Agronomic characteristic analysis

The agricultural yield of T_3_ transgenic and wild-type soybean was analyzed, including parameters such as plant height, branches, pods per plant, seeds per plant and 100-seed weight. Table 1 shows that plant height, seeds per plant, and 100-seed weight per plant of transgenic *MsWRKY11* soybean were higher than those of the wild type, especially OX2.

## Discussion

The WRKY family regulates the expression of genes closely related to biotic and abiotic stresses [52]. The functions of *WRKY* genes have been extensively researched in various plants, including Arabidopsis, rice, soybean, grapevine, sorghum, barley, and maize. However, only a few studies have examined the alfalfa *WRKY* gene family. *Medicago sativa* L. Zhaodong has excellent salt tolerance. The protein sequences of GmWRKY65 and MsWRKY11 have many differences, which might explain the higher level of salt tolerance of Zhaodong alfalfa compared to soybean (S1 Fig). In the present study, we successfully cloned a *WRKY* gene from alfalfa, designated as *MsWRKY11*. The homologous proteins of MsWRKY11 across various plant families were shown in a phylogenetic tree. Salt tolerance genes having high homology with MsWRKY11 in the phylogenetic tree should be further studied in the future.

WRKY TFs have an important role in ABA-responsive signaling networks [22]. In Arabidopsis, *WRKY40*, *WRKY18*, and *WRKY60* function as negative regulators of ABA signaling directly downstream of the ABA receptor ABAR [53]. In the present study, the qRT-PCR experiment showed that expression of *MsWRKY11* was significantly induced under salinity, alkalinity, cold, drought, and ABA stresses. This result demonstrated that *MsWRKY11* is a stress-inducible TF and may function via the ABA-dependent signaling pathway in response to stress.

Salinity is a major factor limiting crop yield worldwide. Each crop has a threshold salinity level, beyond which plant growth decreases as salinity increases [54,55]. The *WRKY* gene family, one of the largest families of TFs, enhances stress tolerance of plants. Overexpression of *RtWRKY1* in *Arabidopsis* confers salt tolerance by regulating plant growth, osmotic balance, Na^+^/K^+^ homeostasis, and the antioxidant system [56]. *DgWRKY3* overexpression in tobacco enhances salt stress tolerance [36]. Despite the current understanding of the functions of *WRKY*, an extensive study on the possible use of *MsWRKY11* for developing soybean cultivars with superior salt tolerance has yet to be conducted. In the present study, the growth of transgenic plants was affected less by salt stress compared to the wild type, and chlorosis of the basal leaves was not detected in transgenic soybean (Fig 6). Chlorophyll content is a vital factor that can be used to determine the quality of plant growth. Overexpression of *ThWRKY4* in Arabidopsis increased the chlorophyll contents significantly in transgenic lines as compared to the control [57], and *JcWRKY* overexpression in tobacco resulted in higher chlorophyll content compared with the wild type under salt stress [35]. Similarly, *MsWRKY11* overexpression in soybean increased the chlorophyll levels in transgenic plants as compared to those in wild-type plants under salt stress conditions (Fig 8). Taken together, these results suggest that *WRKY11* gene expression improves soybean resistance to salt stress. Previous studies reported that overexpression of *DnWRKY11* improved the germination rate, root length, and fresh weight of transgenic tobacco under salt and drought stress when compared with the wild type [58], and expression of *TaWRKY10* increased seed germination rate and root length [59]. These studies were corroborated by our data, which showed higher germination rates in transgenic soybean seeds than in wild-type seeds.

Salt treatment increased the content of ROS in plants, including H_2_O_2_ and O_2_^-^ [60,61]. The main function of CAT is the hydrolysis of H_2_O_2_ into H_2_O and O_2_; SOD eliminates the harmful substances produced by organisms during various metabolic processes. In addition, MDA is toxic to cells and is responsible for the cross-linking polymerization of proteins, nucleic acids, and other living macromolecules. In the present study, relative electrical conductivity and MDA content increased in both transgenic and wild-type plants under salt stress, although the increase was greater in the wild-type plants than in the transgenic lines (Fig 9A and 9B). Salt stress caused increased levels of CAT and SOD in transgenic and wild-type plants, but the increase was higher in transgenic plants (Fig 9C–9F). These data show that the antioxidant ability of transgenic plants was stronger than that of the wild type. Similarly, free proline and soluble sugar content in transgenic and wild-type plants increased under salt stress, with a higher increase in transgenic plants than in the wild type (Fig 9G and 9H). These results indicate that transgenic soybean resisted salt stress by accumulating more soluble protein and proline content. The results presented herein confirm that *MsWRKY11* might reduce ROS levels and then increase salt tolerance in soybean.

In conclusion, MsWRKY11 was identified and characterized as a stress-inducible TF that responds to alkalinity, cold, drought, ABA, and especially salt stress. *MsWRKY11* overexpression improved the salt tolerance in soybean, which further confirmed the role of the *WRKY* gene family in the salt stress response and its potential for engineering soybean lines resistant to saline soil. However, the detailed regulatory mechanisms and functions of the *MsWRKY11* gene in response to other stresses remain to be further investigated.

## Supporting information

S1 Fig Protein sequence analysis of GmWRKY11, MsWRKY11, GmWRKY65, and AtWRKY11. The protein sequence analyzed using DNAMAN.
(DOC)

## References

[1] Kim E, Hwang S, Lee I. SoyNet: a database of co-functional networks for soybean Glycine max. Nucleic Acids Res. 2017; 45: D1082–D1089. doi: 10.1093/nar/gkw704 27492285 10.1093/nar/gkw704 PMC5210602

[2] Niño-Medina G, Muy-Rangel D, Urías-Orona V. Chickpea (Cicer arietinum) and Soybean (Glycine max) Hulls: Byproducts with Potential Use as a Source of High Value-Added Food Products. Waste & Biomass Valor. 2017; 8: 1199–1203.

[3] Qi DH, Lee CF. Influence of soybean biodiesel content on basic properties of biodiesel-diesel blends. JTaiwan Inst Chem E. 2014; 45: 504–507.

[4] Ko KP, Park SK, Yang JJ, Ma SH, Gwack J, Shin A, et al Intake of soy products and other foods and gastric cancer risk: a prospective study. J Epidemiol. 2013; 23: 337–343. doi: 10.2188/jea.JE20120232 23812102 10.2188/jea.JE20120232 PMC3775527

[5] Sulieman S, Ha CV, Esfahani MN, Watanabe Y, Nishiyama R, Pham CTB, et al DT2008: A promising new genetic resource for improved drought tolerance in soybean when solely dependent on symbiotic N_2_ fixation. Biomed Res Int. 2015; 2015: 687213 doi: 10.1155/2015/687213 25685802 10.1155/2015/687213 PMC4299153

[6] Shatters RG, Abdelghany A, Elbagoury O, West SH. Soybean seed deterioration and response to osmotic priming: changes in specific enzyme activities in extracts from dry and germinating seeds. Seed Sci Res. 4 2008.

[7] Rui M, Serralheiro R. Soil Salinity: Effect on Vegetable Crop Growth. Management, Practices to Prevent and Mitigate Soil Salinization. Sci Hortic-Amsterdam. 2017; 3:13.

[8] Stockinger EJ, Gilmour SJ, Thomashow MF. Arabidopsis thaliana CBF1 encodes an AP2 domain-containing transcriptional activator that binds to the C-repeat/DRE, a cis-acting DNA regulatory element that stimulates transcription in response to low temperature and water deficit. Proc Natl Acad Sci U S A. 1997; 94: 1035–1040. 9023378 10.1073/pnas.94.3.1035 PMC19635

[9] Novillo F, Alonso JM, Ecker JR, Salinas J. CBF2/DREB1C is a negative regulator of CBF1/DREB1B and CBF3/DREB1A expression and plays a central role in stress tolerance in Arabidopsis. Proc Natl Acad Sci U S A. 2004; 101: 3985–3990. doi: 10.1073/pnas.0303029101 15004278 10.1073/pnas.0303029101 PMC374356

[10] Kasuga M, Miura S, Shinozaki K, Yamaguchi-Shinozaki K. A combination of the Arabidopsis DREB1A gene and stress-inducible rd29A promoter improved drought- and low-temperature stress tolerance in tobacco by gene transfer. Plant Cell Physiol. 2004; 45: 346 15047884 10.1093/pcp/pch037

[11] Liu Q, Kasuga M, Sakuma Y, Abe H, Miura S, Yamaguchi-Shinozaki K, et al Two transcription factors, DREB1 and DREB2, with an EREBP/AP2 DNA binding domain separate two cellular signal transduction pathways in drought- and low-temperature-responsive gene expression, respectively, in Arabidopsis. Plant Cell. 1998; 10: 1391–1406. 9707537 10.1105/tpc.10.8.1391 PMC144379

[12] Sakuma Y, Maruyama K, Qin F, Osakabe Y, Shinozaki K, Yamaguchi-Shinozaki K. Dual function of an Arabidopsis transcription factor DREB2A in water-stress-responsive and heat-stress-responsive gene expression. Proc Natl Acad Sci U S A. 2006; 103: 18822–18827. doi: 10.1073/pnas.0605639103 17030801 10.1073/pnas.0605639103 PMC1693746

[13] Zhu M, Hu Z, Zhou S, Wang L, Dong T, Pan Y, et al Molecular Characterization of Six Tissue-Specific or Stress-Inducible Genes of NAC Transcription Factor Family in Tomato (Solanum lycopersicum). J Plant Growth Regul. 2014; 33: 730–744.

[14] Wang YX, Liu ZW, Wu ZJ, Li H, Zhuang J. Transcriptome-Wide Identification and Expression Analysis of the NAC Gene Family in Tea Plant [Camellia sinensis (L.) O. Kuntze]. PLoS One. 2016; 11: e0166727 doi: 10.1371/journal.pone.0166727 27855193 10.1371/journal.pone.0166727 PMC5113971

[15] Ji X, Liu G, Liu Y, Zheng L, Nie X, Wang Y. The bZIP protein from Tamarix hispida, ThbZIP1, is ACGT elements binding factor that enhances abiotic stress signaling in transgenic Arabidopsis.BMC Plant Biol. 2013; 13: 151 doi: 10.1186/1471-2229-13-151 24093718 10.1186/1471-2229-13-151 PMC3852707

[16] Katiyar A, Smita S, Lenka SK, Rajwanshi R, Chinnusamy V, Bansal KC. Genome-wide classification and expression analysis of MYB transcription factor families in rice and Arabidopsis. BMC Genomics. 2012; 13: 1–19. doi: 10.1186/1471-2164-13-1 23050870 10.1186/1471-2164-13-544 PMC3542171

[17] Phukan UJ, Jeena GS, Shukla RK. WRKY Transcription Factors: Molecular Regulation and Stress Responses in Plants. Front Plant Sci. 2016; 7: 760 doi: 10.3389/fpls.2016.00760 27375634 10.3389/fpls.2016.00760 PMC4891567

[18] Karkute SG, Gujjar RS, Rai A, Akhtar M, Singh M, Singh B. Genome wide expression analysis of WRKY genes in tomato (Solanum lycopersicum) under drought stress. Plant Gene. 2018; 8–17.

[19] Eulgem T, Somssich IE. Networks of WRKY transcription factors in defense signaling. Curr Opin Plant Biol. 2007; 10: 366–371. doi: 10.1016/j.pbi.2007.04.020 17644023 10.1016/j.pbi.2007.04.020

[20] Rushton PJ, Somssich IE, Ringler P, Shen QJ. WRKY transcription factors. Trends Plant Sci. 2010; 15: 247–258. doi: 10.1016/j.tplants.2010.02.006 20304701 10.1016/j.tplants.2010.02.006

[21] Chi Y, Yang Y, Zhou Y, Zhou J, Fan B, Yu JQ, et al Protein-protein interactions in the regulation of WRKY transcription factors. Mol Plant. 2013; 6: 287–300. doi: 10.1093/mp/sst026 23455420 10.1093/mp/sst026

[22] Rushton DL, Tripathi P, Rabara RC, Lin J, Ringler P, Boken AK, et al WRKY transcription factors: key components in abscisic acid signalling. Plant Biotechnol J. 2012; 10: 2–11. doi: 10.1111/j.1467-7652.2011.00634.x 21696534 10.1111/j.1467-7652.2011.00634.x

[23] Schluttenhofer C, Yuan L. Regulation of specialized metabolism by WRKY transcription factors. Plant Physiol. 2015; 167: 295–306. doi: 10.1104/pp.114.251769 25501946 10.1104/pp.114.251769 PMC4326757

[24] Zhou QY, Tian AG, Zou HF, Xie ZM, Lei G, Huang J, et al Soybean WRKY-type transcription factor genes, GmWRKY13, GmWRKY21, and GmWRKY54, confer differential tolerance to abiotic stresses in transgenic Arabidopsis plants. Plant Biotechnol J. 2008; 6: 486–503. doi: 10.1111/j.1467-7652.2008.00336.x 18384508 10.1111/j.1467-7652.2008.00336.x

[25] Niu CF, Wei W, Zhou QY, Tian AG, Hao YJ, Zhang WK, et al Wheat *WRKY* genes *TaWRKY2* and *TaWRKY19* regulate abiotic stress tolerance in transgenic Arabidopsis plants. Plant Cell Environ. 2012; 35: 1156–1170. doi: 10.1111/j.1365-3040.2012.02480.x 22220579 10.1111/j.1365-3040.2012.02480.x

[26] Hu Y, Chen L, Wang H, Zhang L, Wang F, Yu D. Arabidopsis transcription factor WRKY8 functions antagonistically with its interacting partner VQ9 to modulate salinity stress tolerance. Plant J. 2013; 74: 730–745. doi: 10.1111/tpj.12159 23451802 10.1111/tpj.12159

[27] Cai R, Zhao Y, Wang Y, Lin Y, Peng X, Li Q, et al Overexpression of a maize *WRKY58* gene enhances drought and salt tolerance in transgenic rice. Plant Cell Tiss Org. 2014; 119: 565–577.

[28] Wang F, Hou X, Tang J, Wang Z, Wang S, Jiang F, et al A novel cold-inducible gene from Pak-choi (Brassica campestris ssp. chinensis), *BcWRKY46*, enhances the cold, salt and dehydration stress tolerance in transgenic tobacco. Mol Biol Rep. 2012; 39: 4553–4564. doi: 10.1007/s11033-011-1245-9 21938429 10.1007/s11033-011-1245-9

[29] Jia H, Wang C, Wang F, Liu S, Li G, Guo X, et al *GhWRKY68* reduces resistance to salt and drought in transgenic Nicotiana benthamiana. PLoS One. 2015; 10: e0120646 doi: 10.1371/journal.pone.0120646 25793865 10.1371/journal.pone.0120646 PMC4368093

[30] Rishi A, Sneha S. Antioxidative defense against reactive oxygen species in plants under salt stress. 2017.

[31] Wang F, Liu J, Zhou L, Gang P, Li Z, Zaidi SH, et al Senescence-specific change in ROS scavenging enzyme activities and regulation of various SOD isozymes to ROS levels in *psf* mutant rice leaves. Plant Physiol Biochem. 2016; 109:248 doi: 10.1016/j.plaphy.2016.10.005 27756006 10.1016/j.plaphy.2016.10.005

[32] Fu Y, Guo C, Wu H, Chen C. Arginine decarboxylase ADC2 enhances salt tolerance through increasing ROS scavenging enzyme activity in *Arabidopsis thaliana*. Plant Growth Regul. 2017; (10): 1–11.

[33] Roychoudhury A, Roy C, Sengupta D N. Transgenic tobacco plants overexpressing the heterologous lea, gene Rab16A, from rice during high salt and water deficit display enhanced tolerance to salinity stress. Plant Cell Rep. 2007; 26: 1839–59. doi: 10.1007/s00299-007-0371-2 17554543 10.1007/s00299-007-0371-2

[34] Wu GQ, Liang N, Feng RJ, Zhang JJ. Evaluation of salinity tolerance in seedlings of sugar beet (Beta vulgaris L.) cultivars using proline, soluble sugars and cation accumulation criteria. Acta Physiol Plant. 2013, 35: 2665–2674.

[35] Agarwal P, Dabi M, Sapara KK, Joshi PS, Agarwal PK. Ectopic Expression of JcWRKY Transcription Factor Confers Salinity Tolerance via Salicylic Acid Signaling. Front Plant Sci. 2016; 7. 10.3389/fpls.2016.01541 PMC5065966 27799936

[36] Liu QL, Zhong M, Li S, Pan YZ, Jiang BB, Jia Y, et al Overexpression of a chrysanthemum transcription factor gene, DgWRKY3, in tobacco enhances tolerance to salt stress. Plant Physiol Biochem. 2013; 69: 27–33. doi: 10.1016/j.plaphy.2013.04.016 23707882 10.1016/j.plaphy.2013.04.016

[37] Liang QY, Wu YH, Wang K, Bai ZY, Liu QL, Pan YZ, et al Chrysanthemum *WRKY* gene *DgWRKY5* enhances tolerance to salt stress in transgenic chrysanthemum. Sci Rep. 2017; 7: 4799 doi: 10.1038/s41598-017-05170-x 28684847 10.1038/s41598-017-05170-x PMC5500475

[38] Peng YL, Gao ZW, Gao Y, Liu GF, Sheng LX, et al Eco-physiological characteristics of alfalfa seedlings in response to various mixed salt-alkaline stresses. J Integr Plant Biol. 2008; 50: 29–39. doi: 10.1111/j.1744-7909.2007.00607.x 18666949 10.1111/j.1744-7909.2007.00607.x

[39] Larkin MA, Blackshields G, Brown NP, Chenna R, McGettigan PA, McGettigan H, et al Clustal W and Clustal X version 2.0. Bioinformatics. 2007; 23: 2947–2948. doi: 10.1093/bioinformatics/btm404 17846036 10.1093/bioinformatics/btm404

[40] Kim HJ, Kim MJ, Pak JH, Im HH, Lee DH, Kim KH, et al RNAi-mediated Soybean mosaic virus, (SMV) resistance of a Korean Soybean cultivar. Plant Biotechnology Rep. 2016;10: 257–267.

[41] Kim MJ, Kim HJ, Pak JH, Cho HS, Choi HK, Jung HW, et al Overexpression of *AtSZF2* from Arabidopsis showed enhanced tolerance to salt stress in soybean. Plant Breed Biotech. 2017; 5: 1–15.

[42] Li S, Cong Y, Liu Y, Wang T, Shuai Q, Chen N, et al Optimization of Agrobacterium-Mediated Transformation in Soybean. Front Plant Sci, 2017; 8: 246 doi: 10.3389/fpls.2017.00246 28286512 10.3389/fpls.2017.00246 PMC5323423

[43] Rao X, Huang X, Zhou Z, Lin X. An improvement of the 2ˆ(-delta delta CT) method for quantitative real-time polymerase chain reaction data analysis. Biostat Bioinforma Biomath. 2013; 3: 71 25558171 PMC4280562

[44] Lichtenthaler HK. Chlorophylls and carotenoids: Pigments of photosynthetic biomembranes. Methods Enzymol. 1987; 148: 350–382.

[45] Zhang L, Xi D, Luo L, Meng F, Li Y, Wu CA, et al Cotton *GhMPK2* is involved in multiple signaling pathways and mediates defense responses to pathogen infection and oxidative stress. FEBS J. 2011; 278: 1367–1378. doi: 10.1111/j.1742-4658.2011.08056.x 21338470 10.1111/j.1742-4658.2011.08056.x

[46] Beauchamp C, Fridovich I. Superoxide dismutasee: Improved assays and an assay applied to acrylamide gels. Anal Biochem. 1971; 44: 276–287. 4943714 10.1016/0003-2697(71)90370-8

[47] Hodges DM, Delong JM, Forney CF, Prange RK. Improving the thiobarbituric acid-reactive-substances assay for estimating lipid peroxidation in plant tissues containing anthocyanin and other interfering compounds. Planta. 1999; 207: 604–611. 10.1007/s00425-017-2699-3 28456836

[48] Irigoyen JJ, Einerich DW, Sánchez-Díaz M. Water stress induced changes in concentrations of proline and total soluble sugars in nodulated alfalfa (Medicago sativd) plants. Physiol Plantarum.1992; 84: 55–60.

[49] Lutts S, Kinet JM, Bouharmont J. NaCl impact on somaclonal variation exhibited by tissue culture-derived fertile plants of rice (Oryza sativa L). Journal of Plant Physiology. 1998; 152: 92–103.

[50] Mukherjee SP, Choudhuri MA. Implications of water stress-induced changes in the levels of endogenous ascorbic acid and hydrogen peroxide in Vigna seedlings. Physiologia Plantarum. 1983; 58: 166–170.

[51] Liu F, Pang SJ. Stress tolerance and antioxidant enzymatic activities in the metabolisms of the reactive oxygen species in two intertidal red algae Grateloupia turuturu and Palmaria palmata. Journal of Experimental Marine Biology & Ecology. 2010; 382: 82–87.

[52] Jiang J, Ma S, Ye N, Jiang M, Cao J, Zhang Z, et al WRKY transcription factors in plant responses to stresses. J Integr Plant Biol. 2017; 59: 86–101. doi: 10.1111/jipb.12513 27995748 10.1111/jipb.12513

[53] Shang Y, Yan L, Liu ZQ, Cao Z, Mei C, Xin Q, et al The Mg-Chelatase H Subunit of Arabidopsis Antagonizes a Group of WRKY Transcription Repressors to Relieve ABA-Responsive Genes of Inhibition. Plant Cell. 2010; 22: 1909 doi: 10.1105/tpc.110.073874 20543028 10.1105/tpc.110.073874 PMC2910980

[54] Epstein E, Norlyn JD, Rush DW, Kingsbury RW, Kelley DB, Cunningham GA, et al Saline Culture of Crops: A Genetic Approach. Science. 1980; 210: 399–394. doi: 10.1126/science.210.4468.399 17837407 10.1126/science.210.4468.399

[55] Boyer JS. Plant Productivity and Environment. Science.1982; 218: 443–448. doi: 10.1126/science.218.4571.443 17808529 10.1126/science.218.4571.443

[56] Du C, Zhao P, Zhang H, Li N, Zheng L, Wang Y, et al The Reaumuria trigyna transcription factor RtWRKY1 confers tolerance to salt stress in transgenic Arabidopsis. J Plant Physiol. 2017; 215: 48–58. doi: 10.1016/j.jplph.2017.05.002 28527975 10.1016/j.jplph.2017.05.002

[57] Zheng L, Liu G, Meng X, Liu Y, Ji X, Li Y, et al A *WRKY* gene from Tamarix hispida, *ThWRKY4*, mediates abiotic stress responses by modulating reactive oxygen species and expression of stress-responsive genes. Plant Mol Biol. 2013; 82: 303–320. doi: 10.1007/s11103-013-0063-y 23615900 10.1007/s11103-013-0063-y

[58] Xu XB, Pan Y-Y, Wang CL, Ying QC, Song HM, Wang HZ, et al Overexpression of *DnWRKY11* enhanced salt and drought stress tolerance of transgenic tobacco. Biologia. 2014; 69: 994–1000.

[59] Wang C, Deng P, Chen L, Wang X, Ma H, Hu W, et al A wheat WRKY transcription factor TaWRKY10 confers tolerance to multiple abiotic stresses in transgenic tobacco. PLoS One. 2013; 8: e65120 doi: 10.1371/journal.pone.0065120 23762295 10.1371/journal.pone.0065120 PMC3677898

[60] Miller G, Suzuki N, Ciftci-Yilmaz S, Mittler R. Reactive oxygen species homeostasis and signalling during drought and salinity stresses. Plant Cell Environ. 2010; 33: 453–467. doi: 10.1111/j.1365-3040.2009.02041.x 19712065 10.1111/j.1365-3040.2009.02041.x

[61] Fath A, Bethke PC, Belligni MV, Spiegel YN, Jones RL. Signalling in the cereal aleurone: hormones,reactive oxygen and cell death. New Phytol. 2001; 151: 99–107. 10.1046/j.1469-8137.2001.00153.x 33873372

