# Supplementary figures and images for: Overexpression of the alfalfa WRKY11 gene enhances salt tolerance in soybean
Source: PLoS One. 2018 Feb 21;13(2):e0192382. doi: 10.1371/journal.pone.0192382 (PMC5821330; doi:10.1371/journal.pone.0192382)

S1 Figure


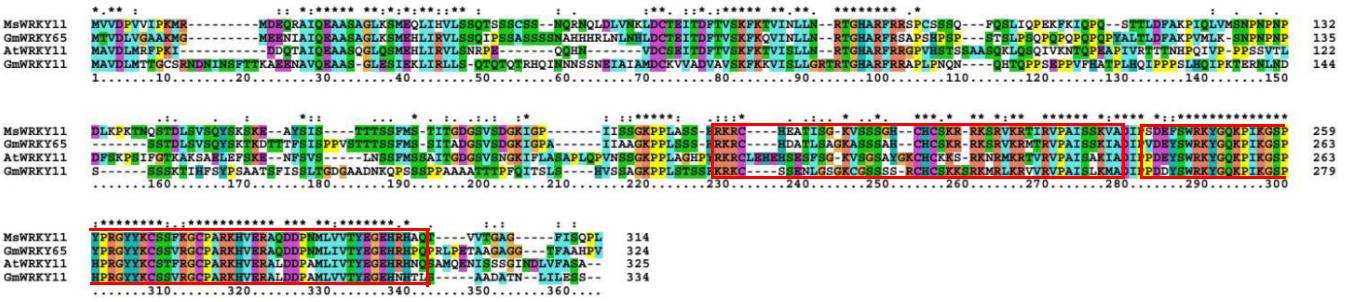

Supplement: S1 Fig — The protein sequence analyzed using DNAMAN. (DOC) [file pone.0192382.s001.doc]
